# The A-Effect and Global Motion

**DOI:** 10.3390/vision3020013

**Published:** 2019-03-28

**Authors:** Pearl S. Guterman, Robert S. Allison

**Affiliations:** Centre for Vision Research, York University, Toronto, ON M3J 1P3, Canada

**Keywords:** vision, A-effect, vection, subjective visual vertical, tilt, orientation, self-motion, gravity, motion, motion parallax

## Abstract

When the head is tilted, an objectively vertical line viewed in isolation is typically perceived as tilted. We explored whether this shift also occurs when viewing global motion displays perceived as either object-motion or self-motion. Observers stood and lay left side down while viewing (1) a static line, (2) a random-dot display of 2-D (planar) motion or (3) a random-dot display of 3-D (volumetric) global motion. On each trial, the line orientation or motion direction were tilted from the gravitational vertical and observers indicated whether the tilt was clockwise or counter-clockwise from the perceived vertical. Psychometric functions were fit to the data and shifts in the point of subjective verticality (PSV) were measured. When the whole body was tilted, the perceived tilt of both a static line and the direction of optic flow were biased in the direction of the body tilt, demonstrating the so-called A-effect. However, we found significantly larger shifts for the static line than volumetric global motion as well as larger shifts for volumetric displays than planar displays. The A-effect was larger when the motion was experienced as self-motion compared to when it was experienced as object-motion. Discrimination thresholds were also more precise in the self-motion compared to object-motion conditions. Different magnitude A-effects for the line and motion conditions—and for object and self-motion—may be due to differences in combining of idiotropic (body) and vestibular signals, particularly so in the case of vection which occurs despite visual-vestibular conflict.

## 1. Introduction

Many activities in our daily life such as walking, riding a bike and even sitting still, rely on our ability to control our equilibrium. Our sense of orientation relative to gravity helps us maintain both static equilibrium (postural balance) when we are still and dynamic equilibrium when we are in motion [1,2]. Verticality or the direction of ‘up’ can be derived from visual cues in our environment such as the polarity of an object [3,4,5]—e.g., knowing that a tree trunk is rooted in the ground—and from non-visual cues which relay information about our body orientation in space. Non-visual self-orientation cues include those from the vestibular system, which is sensitive to angular and linear accelerations (including gravity) and thus senses static or dynamic head tilt [2,6], as well as somaesthetic cues from proprioception, interoception (e.g., the gut and baroreceptors [7,8]) and exteroception (e.g., touch and pressure—Horak et al. [9]). We need to make judgements both of our own orientation and of objects in our environment. Several studies have suggested that judgements our own orientation with respect to gravity do not necessarily determine our judgements of the orientation of other objects [10,11,12].

Observers lying on their side (roll-tilted) in the dark make appreciable systematic errors when asked to estimate the orientation of a line or other objects relative to gravitational vertical [10,13,14]. Aubert [13] was the first to observe that a vertical luminous line appears tilted when the head is roll-tilted in the dark. Müller [15] examined this effect and found that a vertical line appears tilted in the same direction as the head for head tilts greater than 60° (“A-effect”) and in the opposite direction for smaller head tilts (“E-effect”). Kaptein & Van Gisbergen [10] reported that E-effects are also found again at larger angles (> 135°). While A and E effects could reflect an over and underestimation of self-tilt, respectively, observers with modest errors in their judgements of self-tilt (relative to gravity) still tend to make gross systematic errors in judging external object tilt [10,12,16]. Mittelstaedt [12] suggested that judgements of the visual vertical are biased toward alignment with an egocentric frame of reference (i.e., toward the long axis of the body which he called the ‘idiotropic vector’) and he developed an influential model to explain the data [17]. In this model, consistent with their data, judgements of body-orientation are not subject to these idiotropic biases [10,12,17].

If objectively vertically-oriented objects appear to be tilted to the tilted observer, then one might expect that objects moving vertically should appear to move along tilted paths. It is unclear if motion is subject to the same A-effect observed with static lines. De Vrijer et al. [18] compared illusory tilt in the direction of planar motion with that in static line displays in tilted observers. They found a similar pattern of systematic errors for the line and motion displays and so concluded that orientation judgments of both static tilt and motion involved a common spatial reference frame and a shared computational strategy. Their brief motion displays contained random visual noise to minimize local directional cues. In contrast, as we move through a static environment in everyday life, the optic flow is coherent except for the motion parallax associated with the depth structure of the world. It is unknown whether a similar pattern of tilt estimation errors would be found when observers are presented with coherent motion and additional visual cues such as motion parallax.

Motion also provides an interesting case in the context of the above-mentioned distinction between reference frames used in judgements of self-orientation and object orientation. Visual motion can be produced both by motion of objects in the environment and by self-motion. Visual information is a powerful self-motion cue and optic flow generated by simulated visual scene motion in stationary observers, can induce an illusion of self-motion known as vection [19]. Given that allocentric and egocentric reference frames seem to play different roles in determining object and self-orientation, we hypothesized that tilted observers may exhibit different biases for judgements of the direction of vection than judgments of the motion of external objects [20]. To test this, we assessed whether object and self-motion are subject to the same A-effect observed with static lines.

In the present experiments, we compared the influence of whole-body tilt on the perceived orientation of a line with the apparent tilt in the direction of various types of global motion (Experiment 1). We also compared perceived motion direction relative to gravitational up when the motion was perceived as external scene motion with perceived motion direction during vection in which the scene appears stationary and visual motion is attributed to the self (Experiment 2). We hypothesized that for recumbent observers, (1) there would be a consistent A-effect for global motion, (2) consistent with De Vrijer et al. [18], this effect would be similar in magnitude as found for static stimuli and (3) the A-effect would be stronger for illusory self-motion, which may not be subject to an idiotropic bias, compared to motion that is perceived as external to the self.

## 2. Experiment 1

Upright and tilted observers judged the tilt of a static line and the direction of both coherent planar and volumetric flow to examine whether systematic errors in tilt were affected by the presence of motion parallax.

### 2.1. Materials and Methods

#### 2.1.1. Subjects

Twenty observers (eight males, twelve females; mean age = 26.7, SD = 5.5) participated. Reliable psychometric functions could not be fit for two observers leaving data for eighteen observers in the analysis. All subjects had normal or corrected-to-normal vision and no reported vestibular impairment. Written informed consent was obtained in accordance with a protocol approved by the York University Research Ethics Board and that conformed to the 1964 Declaration of Helsinki (certificate e2012-012).

#### 2.1.2. Apparatus

**Figure 1:**
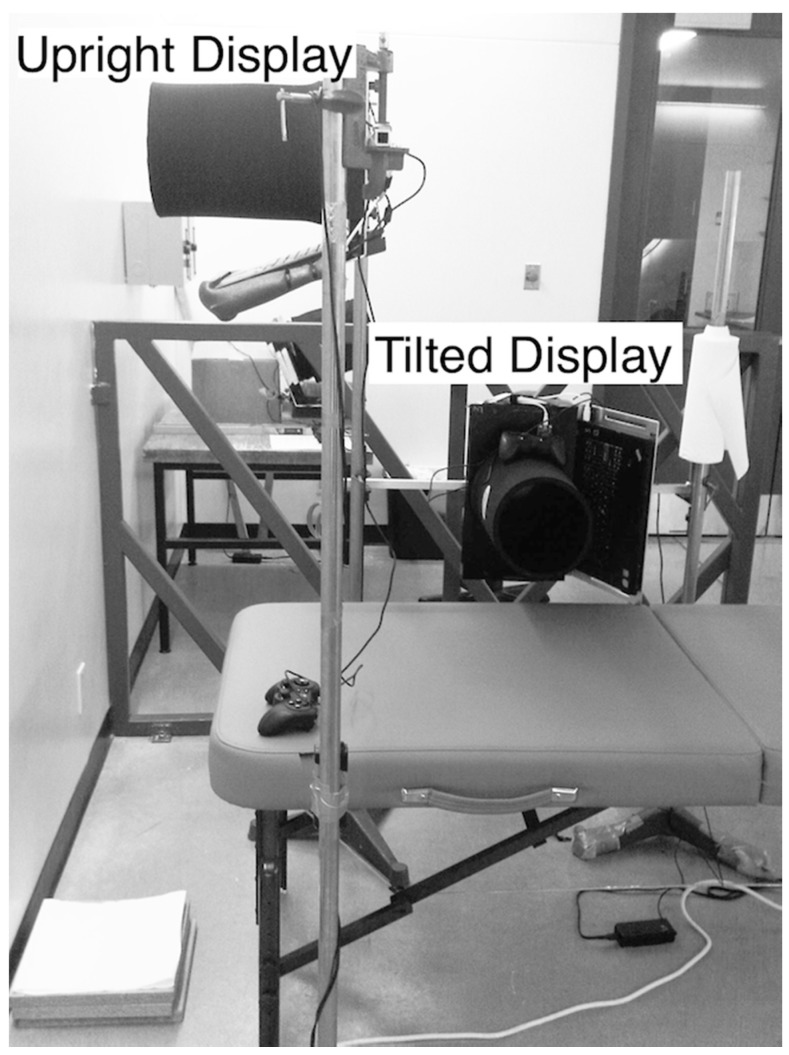
Photograph of the apparatus for the upright (standing) and tilted (lying on side) posture. Foot blocks (bottom left) and a foam headrest (not pictured) were used for height adjustment and support.

Subjects stood upright on stable foot blocks or were supported on a foam mattress with a headrest at a full-body tilt of −90° (left side down) about the naso-occipital (roll) axis. Care was taken to align and centre the head with the computer screen. The stimuli were generated on an IBM Lenovo T61p 15.4-in. TFT laptop using custom Python software (version 2.7, http://python.org, Python Software Foundation, Wilmington, DE, USA) and open-source Pyglet 1.1.4 libraries (http://pyglet.org). Visual displays were generated with a resolution of 1280 (horizontal) x 800 (vertical) pixels and refresh rate of 60 Hz. The laptop was mounted to a rigid frame with the screen frontal-parallel to the subject. Extraneous stimuli were masked using a viewing tube, cloth shroud and a matt-black opaque aperture panel offset 1.5 cm from the screen. This aperture system set the observer-to-screen distance of 30 cm and the visual angle of 39°. Responses were recorded using a Logitech R Dual Action Gamepad (see Figure 1). Subjects wore 3M 1100 earplugs to attenuate extraneous auditory orientation cues.

#### 2.1.3. Visual Displays

The displays were a static line that spanned the display (39° × 0.2°) and two types of global motion dot stimuli. Both the line and dots were blue (16.72 cd/m^2^) on a black background (0.64 cd/m^2^). The motion conditions included planar (2-D) and volumetric (3-D) optic flow. The motion displays contained randomly-distributed dots in a computer-generated world. The dots modelled spheres in the rendered scene with a simulated radius of 7.5 cm. The motion was produced by translating the virtual camera parallel to the screen to produce a lamellar flow pattern that moved upward in head-centric coordinates (for 0° tilt conditions) with a simulated speed of 1.33 m/s. When any dot moved beyond the field of view (off screen), it was redrawn at the same original horizontal and depth coordinates on the opposite side of the virtual scene. The line and motion axes were tilted by rotating the virtual camera, which produced the viewpoint used to render the scene.

**Figure 2:**
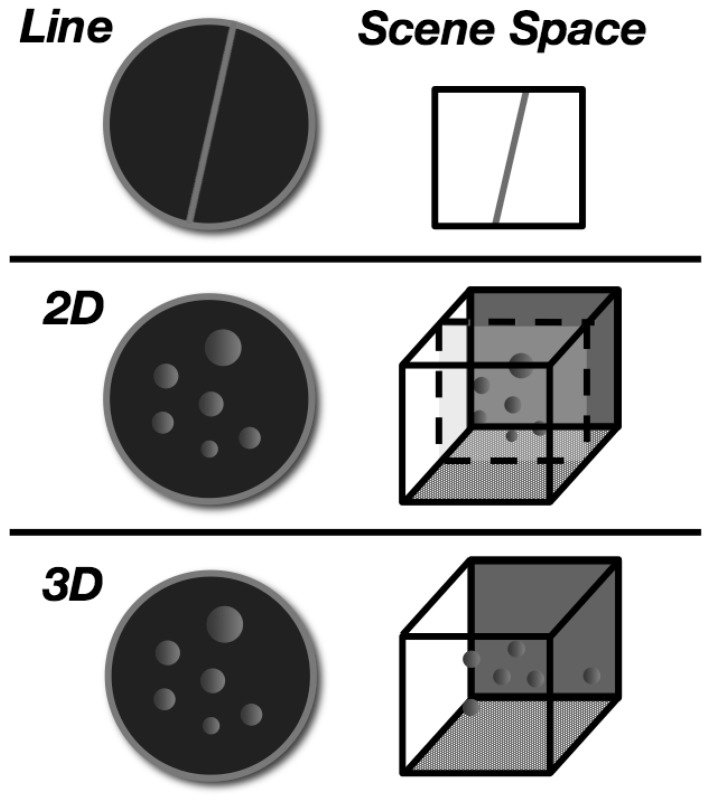
Illustration depicting the virtual depth of the line segment and dots in the virtual scene space shown on the displays. The top of the image shows the line segment that was drawn in 2-D space and extended across the entire display. The middle depicts the 2-D lamellar flow. The dots were drawn in different sizes on a plane positioned at the middle of the depth range (15 m) and therefore had no motion parallax. The bottom image shows the 3-D lamellar flow stimulus with dots drawn along the full virtual depth range (0.1-30 m). Static images of the 2-D and 3-D stimuli looked the same but in motion only the 3-D stimulus provided the visual cue of motion parallax.

**Figure 3:**
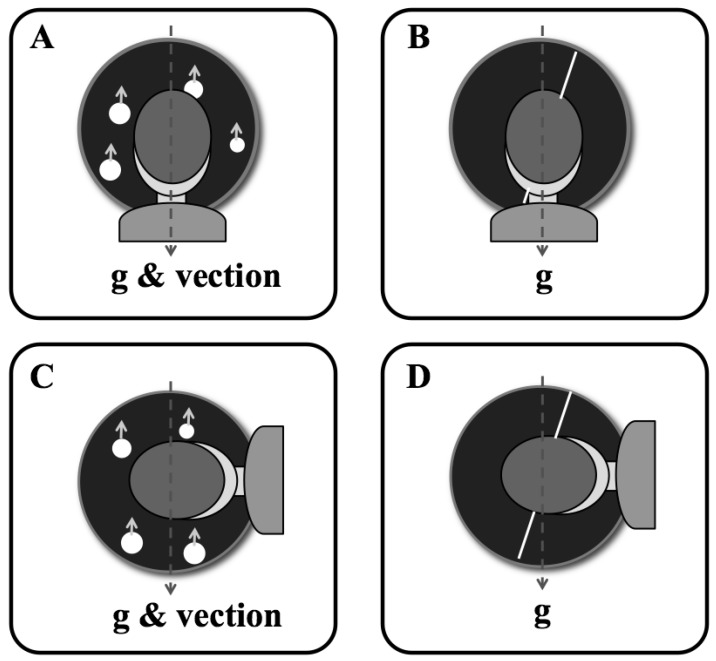
Visual representation of the head orientations, object orientations and object-motion directions relative to the direction of gravity. Perceived self-motion (vection) direction predicted in Experiment 2 is also shown. The dot and line stimuli are shown as presented to upright (**A**,**B**) and tilted participants with the body left side down (**C**,**D**). The arrows attached to the dots represent the upward motion direction of the dots, which in the self-motion condition (in Experiment 2) would be perceived as downward self-motion (if the direction were perceived veridically).

For the volumetric flow displays, there was a simulated cloud of 5000 dots that extended 30 m along the depth or visual axis. The apparent velocity of these dots was a function of their distance and relative angular displacement at the camera viewpoint, providing the depth cue of motion parallax (the displays were 3D computer graphics renderings but were not stereoscopically presented). For the planar flow displays, the dots were sized to appear to be at depths ranging from 0.1 (the near clipping plane) to 30 m but were actually drawn at the middle of the depth range (at 15 m) to form a single moving plane of dots. Due to these different depth projections, the volumetric and planar stimuli had the same appearance when static but not while in motion. Effectively, the volumetric displays simulated a linear flow field that would be consistent with real observer translation, whereas the planar displays produced the impression of motion relative to a wall with dot wallpaper. Figure 2 shows a schematic of the stimuli and the depth of the objects in the scene space. Figure 3 illustrates the orientation of the static line and the direction of lamellar flow, relative to the direction of gravity, for the upright and tilted postures.

#### 2.1.4. Design

There were two independent variables of interest: body orientation (0° or −90° tilt) and stimulus type (line, planar or volumetric lamellar flow). In preliminary experiments [20] we found similar magnitude A-effects for both left and right recumbent posture and both horizontal (leftward or rightward) and vertical (upward or downward) stimulus motion. Consistent with the main experiment reported here, the points of subjective verticality (PSV) were biased in the direction of the head tilt and bias was significantly larger for the static line than 3D global motion. Given this, we restricted testing to upward motion and 0°/−90° body orientation to allow for sufficient sampling of the psychometric functions.

The magnitude of the A-effect was measured for each of the stimulus types by comparing the point of subjective verticality (PSV) in 90° and 0° orientations. In each condition, the PSV was estimated by fitting psychometric functions to perceived orientation as the stimulus tilt was varied according to the method of constant stimuli. Each stimulus was presented for 500 ms. The static line or direction of motion was tilted 0°, ±10°, ±20° and ±30° from the gravitational vertical. Each of the 42 factorial combinations (2 body tilts × 3 stimulus types × 7 stimulus tilts) was repeated 20 times for a total of 840 trials per subject. Trials were blocked by posture and pseudo-randomly ordered to avoid immediate repetition of the same condition. The blocks were ordered using a counterbalanced design.

#### 2.1.5. Procedure

Observers were told to look casually about the screen while attending to the direction of line or dot motion. While viewing the displays in the upright and tilted postures, the head was aligned with the trunk of the body and the legs were extended. The trials began after at least 60 s in the given posture.

All of the displays were followed by a black, blank screen, during which observers pressed one of two shoulder buttons on the gamepad to indicate whether the stimulus (or its direction of motion) appeared to be tilted clockwise or counter-clockwise from the gravitational vertical (e.g., motion direction for dots that appeared to start bottom-left and move to the top-right would be judged as clockwise). As this was a two-alternative forced choice (2-AFC) procedure, observers were instructed to select one of these button options, even if the line or motion axis did not appear to be tilted.

#### 2.1.6. Data Analysis

Data analyses were performed with RStudio (version 0.98.1103, RStudio, Boston, MA, USA). Individual psychometric functions were fit for each observer and combination of body orientation and stimulus type. The dichotomous responses data (CCW versus CW responses) were fitted to a psychometric curve as a function of visual stimulus tilt using the R package psyphy (https://cran.r-project.org/web/packages/psyphy/, Ken Knoblauch, University of Lyon, France). The data were fit to a sigmoidal psychometric curve defined by a logit link function using maximum likelihood estimation and allowing for variation of both upper and lower asymptotes [21]. This procedure provides a prediction of the expected proportion of clockwise responses as function of stimulus tilt for each condition and subject, which was compared to the actual proportion of clockwise responses obtained at each tilt angle to verify the fits.

The subjective visual vertical was defined by the Point of Subjective Verticality (PSV) and is the fitted angle at which the proportion of clockwise responses was 0.5 (or 50%). The slope of the psychometric function was used to determine the discrimination threshold or just noticeable difference (JND). The inverse of the slope of the logit fit was used as the measure of JND, which corresponds to the change in tilt from the PSV where the fitted proportion of clockwise responses was 0.73 (or 0.27 for a JND change in the other direction).

The resulting PSV and JND data were analysed using linear mixed effects (function lme from the R package *nmle*, http://cran.r-project.org/web/packages/nlme/) regression models, with fixed effects (posture, stimulus type) and a random effect to model inter-subject variability. Outlying points were identified through regression diagnostics and visual inspection of the response measures. Based on these tests, there were 5 data points (from 3 subjects) identified as outlying and they were removed from the dataset. Both JND and PSV estimates for the fitted curve were removed from the analysis for these points (5 out of 108 JND/PSV pairs for 18 observers x 6 conditions were excluded in total) and there was no substitution of deleted data points as mixed effects models are robust to missing data; the pattern of results, including which effects were significant, was similar when these data were included. The final regression models were selected by adopting stepwise selection using Akaike’s Information Criterion (AIC). A goodness-of-fit test based on the analysis of deviance was used to evaluate the fit of the model. Planned comparisons of the A-effect magnitudes were performed with linear contrasts using Wald tests with adjustments to control family-wise error.

### 2.2. Results and Discussion

**Figure 4:**
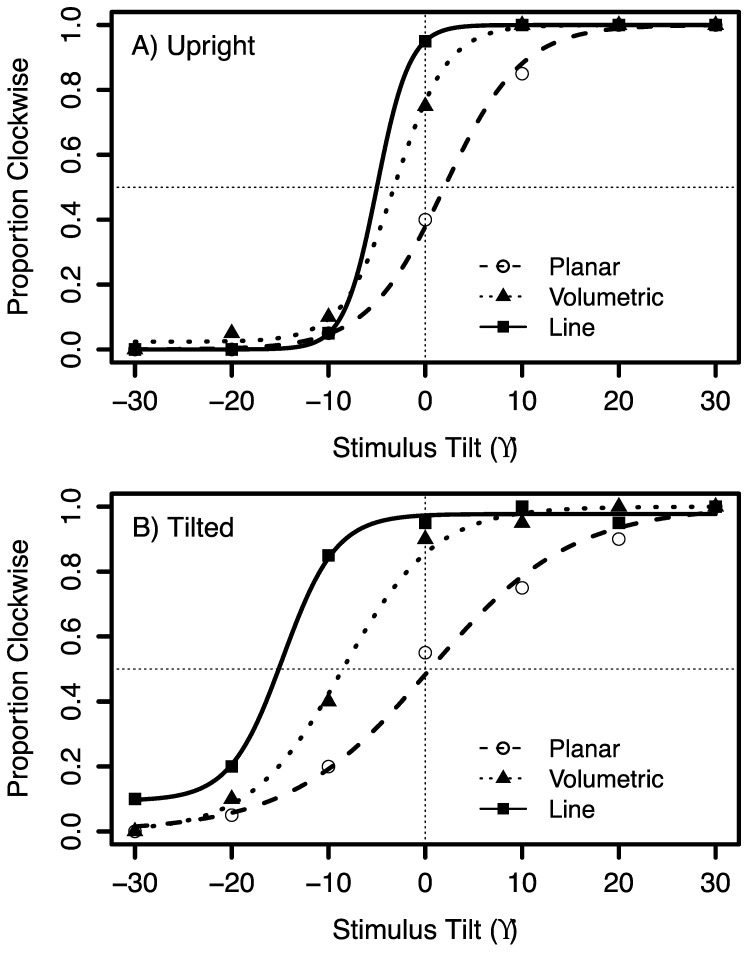
Psychometric functions showing the proportion of times that the stimulus was perceived as tilted in the clockwise direction relative to the gravity vector. The fitted psychometric functions from a single subject for the (**A**) “Upright” and (**B**) “Tilted” head orientations. Planar flow, volumetric flow and the line are represented by an open circle, filled triangle and filled square, respectively.

**Figure 5:**
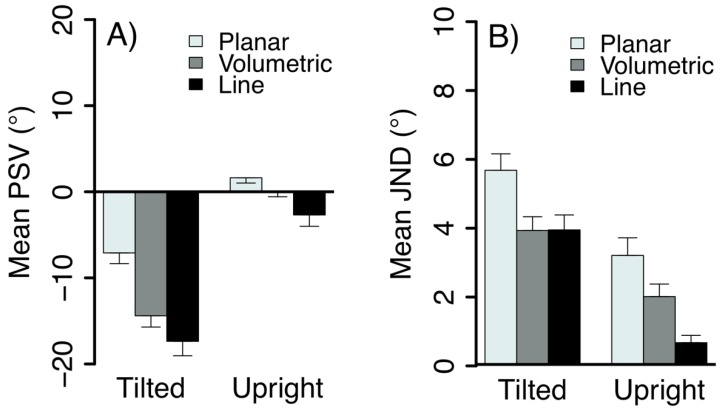
The mean (**A**) Point of subjective verticality (PSV) and (**B**) Just noticeable difference (JND) averaged across observers as a function of head tilt and stimulus type (±1 standard error of the mean (SEM)). A lower JND represents greater certainty or precision in judging the stimulus tilt.

Psychometric functions were obtained by fitting the proportion of clockwise responses as a function of body and stimulus tilt. Figure 4 shows example responses from one subject for the line and global motion in the upright and tilted body orientation and Figure 5 shows the mean results for the fitted parameters of the psychometric functions. When the body was upright the responses were close to veridical. Tilting the body resulted in significant shifts in the perceived vertical in the direction of the body tilt compared to the upright condition, F(1,80) = 95.23, *p* < 0.0001. The stimulus type also had a significant influence on tilt judgments, F(2,80) = 26.38, *p* < 0.0001 and an interaction with body tilt, F(2,80) = 5.48, *p* = 0.0059. The shifts in the PSV when observers were tilted were smaller for the motion conditions than for the line, with a significantly smaller shift for planar flow, compared to volumetric flow, *t*(17) = −5.17, *p* < 0.0001 and the line, *t*(17) = −7.27, *p* < 0.0001. There was also a significant PSV difference between the volumetric flow and the line condition, *t*(17) = −2.10, *p* = 0.0493. Figure 4A,B and Figure 5A show the psychometric functions and mean PSV in degrees for the tilted and upright body orientations when observers viewed the line, planar and volumetric flow.

The mean just noticeable differences (JNDs) are shown in Figure 5B. Overall, observers had significantly higher tilt discrimination thresholds when they were tilted than when upright, F (1,82) = 60.58, *p* < 0.0001. This finding was consistent with previous reports of increased variability in subjective visual vertical settings when observers are tilted [16,22]. These thresholds were found to differ across the stimulus types, F(2,82) = 14.89, *p* < 0.0001; the interaction term was not significant (*p* > 0.05). Observers had higher discrimination thresholds for the planar flow stimulus than for both volumetric flow, *t*(17) = −3.75, *p* = 0.0016 and the line, *t*(17) = −5.43, *p* < 0.0001, regardless of body tilt. There was no significant difference in the JNDs between the volumetric flow and line stimuli, *t*(17) = −1.66, *p* = 0.1153.

Given the differences in discrimination thresholds, we considered that the shifts in the PSV might reflect the choice of psychometric procedure. That is, the differences between conditions might have reflected an increasing regression toward the mean of the stimulus set with more imprecise stimuli. Therefore, we repeated the PSV estimates using an adaptive staircase procedure (*N* = 8, mean age = 26.0, SD = 6.82). As in the main experiment, the observer’s task was to discriminate CW from CCW tilt of the line or motion. For each observer, twelve interleaved staircases followed a 1-up 1-down rule to converge on the PSV (2 body postures × 3 stimulus types × 2 starting tilts, CW and CCW). The results were qualitatively and quantitatively consistent with the findings from the method of constants procedure in the main experiment. Repeated-measures ANOVA indicated a significant effect of stimulus type (F(2,42) = 4.92, *p* =0.0120) and, as in the main experiment, mean PSV in the tilted orientation was largest in the line, followed by the 3D flow and then the 2D flow condition (M = 25.05, 17.46 and 12.36°, respectively).

**Figure 6:**
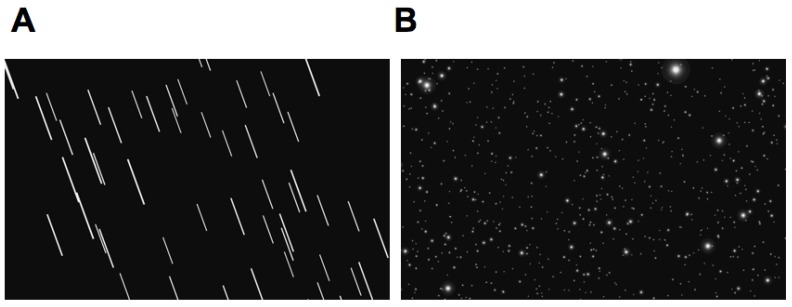
Screenshots of (**A**) the multiple-line stimulus and (**B**) dot stimulus.

In another control experiment (*N* = 16, mean age = 26.75, SD = 5.13), we compared observer tilt judgments for single and multiple line (see Figure 6A) displays that had the same mean luminance as the dot displays. We found no significant differences between these conditions, suggesting that the difference in body tilt-induced bias between the line and motion stimuli was not due to differences in element number, density, eccentricity or luminance. We used a single velocity in the current experiment so can only speculate on how stimulus speed effects the bias. Human direction discrimination threshold functions for dot stimuli are u-shaped with velocity and constant over a large range of velocities especially for large displays [23], so we expect our results to generalize well across speed. However, as a reviewer pointed out, very fast motion may be perceived as motion streaks [24] and thus might be expected to give similar PSV to our multiple line stimuli.

Our finding of a stimulus-dependent A-effect seems inconsistent with the proposal that systematic errors in visual tilt judgments are due to misestimates of self-tilt since errors in estimating body tilt should affect the frame of reference for visual tilt estimates and thus should affect all tilt judgments equally. Rather, we found significant differences in the tilt estimates for the two motion conditions of planar and volumetric flow; these stimuli were the same pictorially and only differed by the addition of motion parallax in the volumetric flow condition. Thus, tilt estimates were not the same for motion in general and may reflect differences in processing different types of global motion. Furthermore, the tilt biases differed between motion orientation and line orientation judgments.

The perception of subjective visual vertical typically involves the integration of visual, vestibular and other cues to orientation [4,25,26]. In the present experiments, the motion tilt and static tilt signals were informative about head-centric orientation of the stimulus. However, unlike the classic rod-and-frame stimulus and similar visual stimuli with frame cues [3,4,27], there were no visual frame or other orienting features in the present experiment and thus the visual stimuli themselves did not provide informative cues to gravitational vertical. Nevertheless, there is bias in judgements of the verticality of these signals toward the head (idiotropic) orientation and this is more pronounced in the line compared to motion conditions. A control experiment using an adaptive staircase procedure produced results that were consistent with these findings and thus these conclusions are not dependent on choice of psychophysical procedure.

## 3. Experiment 2

An optic flow display like those used in Experiment 1 can be perceived as external scene motion or as vection, in which the scene appears stationary and the motion is attributed to the self. In vection the external world is typically perceived as stationary, constant and rigid, so it is possible that cues to the visual vertical are treated differently than when the visual world is perceived as changing and dynamic relative to a stable self. If so, differences in the point of subjective verticality (PSV) for perceived object and self-motion might reflect a change in sensory weighting for vestibular signals during vection. Alternatively, given that previous studies have shown that judgements of tilt of visual objects are subject to larger A-effects than judgements of body tilt [11,12], we might expect that vection, which is attributed to the self, might be less susceptible to A effects than object-motion.

### 3.1. Methods

Eight observers (4 males, 4 females; mean age = 30.3, SD = 8.65) participated in Experiment 2. All subjects had normal or corrected-to-normal vision and no reported vestibular impairment. All had prior experience with judging illusions of self-motion in a laboratory setting.

The apparatus, stimuli and procedures were the same as in Experiment 1 with the following exceptions. First, the number of randomly-distributed dots portrayed was increased to 8500. Second, all stimuli were displays of volumetric flow. The duration of these displays was experimentally controlled to promote either the perception of “external” motion (0.5 s) or “self” motion (20 s). Note that no observers reported experiencing vection in the “external” trials and vection latencies in the self-motion trials were greater than 0.5 s. There were 28 factorial combinations (2 body tilts x 2 stimulus durations x 7 stimulus tilts), each repeated 8 times for a total of 224 trials.

Self-motion trials were preceded by an auditory bell prompt to notify observers that they should attend to their perception of self-motion. During these self-motion trials, observers indicated if and when they experienced their first sensation of self-motion (i.e., vection onset) by briefly pressing one of the shoulder buttons on the gamepad.

As in experiment 1, observers were required to report the tilt direction of the motion display. Observers were told to attend to the angle of the trajectory and disregard the direction (up or down) of the motion (Figure 3, note the expected direction of vection is downward, opposite to the upward motion of the dots in the display); however, for the self-motion trials, if vection was not experienced observers were to press one of the front-facing circular buttons on the gamepad rather than indicating clockwise or counter-clockwise.

### 3.2. Results and Discussion

After regression diagnostics, there was one subject excluded from the analysis. All of the observers experienced vection during the self-motion trials. Vection occurred in 841 out of 896 self-motion trials or approximately 93.86% of the total responses. Self-motion trials in which vection was not experienced were not included in the computation of the PSVs and JNDs. The vection responses from one observer indicated the perceived direction of self-motion rather than the tilt of the motion axis, so their responses for those trials were appropriately reversed (the observer confirmed they made this interpretation after the session). Mean vection latency across observers during these trials was 5.78 ± 4.50 s.

**Figure 7:**
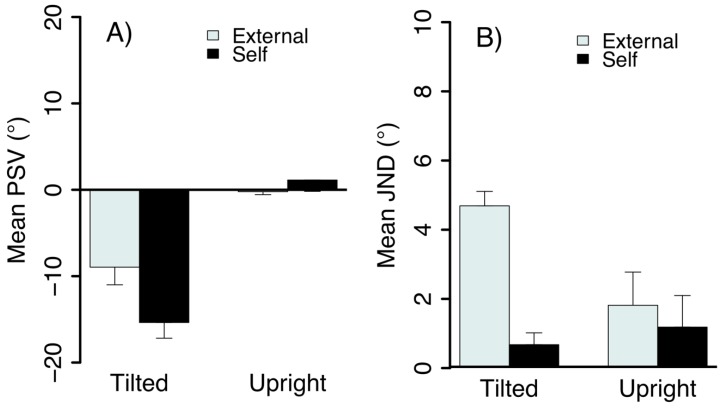
The mean (**A**) PSV and (**B**) JND as a function of body tilt and stimulus duration (±1 SEM).

The mean PSV for the two postures when stimulus motion was perceived as object-motion and self-motion are shown in Figure 7A. Little bias was observed when upright but bias increased significantly when the head was tilted, F(1,15) = 20.92, *p* = 0.0004. This result is an A-effect in that the tilt bias was in the direction of the head tilt. There was also a significant effect of stimulus type (duration) on tilt biases, F(1,15) = 11.29, *p* = 0.0043; shifts in the PSV were greater when the motion was perceived as self-motion compared to scene motion. The interaction between head tilt and stimulus duration was significant, F(1,15) = 7.98, *p* = 0.0128, with greater PSV shifts between external- and self-motion conditions when observers were tilted, *t*(6) = −3.36, *p* = 0.0152.

The mean JNDs are shown in Figure 7B. There was a significant effect on the JND of posture, F(1,15) = 11.18, *p* =0.0044, perceived motion type (object- and self-motion), F(1,15) = 23.60, *p* = 0.0002 and an interaction between these factors, F(1,15) = 7.50, *p* = 0.0152. Tilted observers had significantly lower tilt thresholds when the motion was perceived as self-motion than external motion, *t*(6) = −4.86, *p* = 0.0028. As illustrated in Figure 7, tilting observers resulted in larger systematic errors when observers judged the perceived direction of self-motion compared to object-motion, despite greater precision in the former condition.

In sum, we report the novel finding of an A-effect for visually-induced self-motion. The bias in PSVs with head tilt was significantly greater when global motion was perceived as self-motion than as external motion. We also found a significant difference in the JNDs for the perceived direction of external motion and self-motion relative to gravity. As in Experiment 1, the motion signals themselves were not informative as to the direction of gravitational upright but only about the head centric direction of the stimulus. Nevertheless, there appears to be a bias in judgements of the verticality of these signals toward the head (idiotropic) upright and this is more pronounced in the self-motion conditions. These results would be consistent with the position that idiothetic visual signals are more heavily weighted than vestibular cues to motion verticality during self-motion. However, the JNDs indicated that tilted observers were more precise in their tilt judgments when perceiving self-motion than external motion. It is not clear whether the greater precision reflects more inherent sensitivity for judgements of self-motion or simply averaging over the longer observation time. Adaptation to motion during the long duration stimulus is expected and it has been proposed that this reduces vection [23,28,29]. Adaptation might be expected to weaken the motion signal and increase JND; however, human direction discrimination thresholds are constant over a wide range of velocities [30] and we found a decrease in JND rather than an increase.

## 4. General Discussion

We investigated the effects of head tilt on the perceived direction of global motion relative to gravity, when the motion was experienced as external to the self (object-motion) or induced the sensation of self-motion (vection). When subjects viewed a static line or motion displays while their body was tilted, the line or axis of motion—along which dots moved visually upward—were perceived to be tilted in the direction of the body tilt. These results are consistent with those of De Vrijer et al. [18] and demonstrate that the A-effect can occur with both static and moving visual stimuli. In addition, we found significantly larger PSV shifts and greater precision in judging tilt of a static line than for the motion stimuli (and the shift for volumetric flow was in turn greater than for planar flow). While the difference in tilt bias between the line stimulus and volumetric global motion was modest, in contrast to De Vrijer et al. [18], there were large significant differences in bias between the planar flow and line condition in tilted subjects. The dependence of the perceived tilt on the type of stimulus motion, suggests that the perceived verticality of these stimuli might rely on another underlying factor.

As well as these differences in bias there were differences in precision between the stimuli. The greater precision for the line and volumetric flow may reflect that these stimuli contained more reliable visual cues to the head centric stimulus orientation than the planar flow. In the case of the line, subjects were presented with a salient tilt signal that did not require tracking the direction of motion across the retina. Unlike the planar flow, the volumetric stimulus included the cue of motion parallax due to perspective projection, which can also aid in determining the direction of the motion. Thus, it is not surprising that the precision of the judgements varied across the stimuli but these differences in precision cannot explain the increased bias in subjective visual vertical on their own. Judgements of verticality need to account for head and eye orientation to convert tilt in a head/eye centric frame of reference to allocentric coordinates.

### 4.1. Coordinates, Frames of Reference and Priors

In everyday situations, a visual stimulus not only has an orientation with respect to vertical but can also provide a cue to vertical itself. However, in the present experiments there were no reliable cues to orientation in the test stimuli themselves. While there are biases, perceptual norms or priors toward cardinal direction in perceived motion and tilt [31] these should affect all of our test stimuli. The A-effect has traditionally been explained as a bias or norm for upright posture. Recent investigators have re-posed this explanation in Bayesian terms. For instance, MacNeilage et al. [32] have proposed that the brain incorporates a joint prior that favours 1-g acceleration and upright posture. When this prior is combined with the otolith signal the posterior probability is biased toward the upright, leading to the A-effect. If visual and other cues to true orientation are added the influence of the prior should be reduced and the A-effect reduced. Presence of additional visual information can affect the illusory tilt of a vertical test line in tilted observers. Aubert [13] found the illusory tilt in the dark was greatly reduced or eliminated when the room lights were turned on. Visual frame, polarity and motion cues can indicate the visual upright and influence the judgements [4,26]. Thus, a rectangular frame or even a single line can influence the settings by providing a visual cue to vertical or horizontal. In our gravitationally-constrained world, motion along the cardinal vertical and horizontal directions is typical. Thus, the coherent motion in these displays could have provided a visual cue to vertical, particularly for the volumetric flow condition. Mittelstaedt [33] measured the subjective vertical in tilted observers viewing a number of textured stimuli including an image of a polarized natural environment. If these stimuli were presented at a fixed orientation with a superimposed luminous line then the visual surround should have biased the settings as previous investigators found, more so for the more polarized stimuli.

However, in the present case, the test stimulus was shown in isolation and its tilt judged. Analogously, Mittelstaedt also had conditions where the subject adjusted the orientation of the entire visual scene itself to upright. In this case, the bias found was the same regardless of the type of scene and similar to the line in isolation. Thus, we do not expect the visual orientation cues in the stimulus itself to affect the PSV. However, Witkin and Asch [34] reported that the precision of the settings was much worse in the presence of a simple frame than a furnished room. The volumetric motion and the line stimuli are less ambiguous than the planar motion stimulus with its relatively impoverished visual orientation cues and this might explain why the JNDs were larger for the planar motion stimulus.

We also report that the A-effect occurs not only for motion perceived as external to the body but also for motion perceived as self-motion. Typically, judgements of body orientation show smaller (or no) Aubert effects compared to judgements of object orientation. Thus, we had predicted that by analogy judgements of self-motion direction might be less affected by body tilt than judgements of object-motion direction. Clearly this was not the case as there were generally larger tilt-induced PSV shifts when subjects experienced vection compared to object (volumetric) motion. A lack of vestibular signals consistent with the presented visual motion—as in the self-motion condition—could result in the favouring of the idiotropic bias over the vestibular signal, as the more reliable directional signal. Sensory signals from the visual and vestibular system must be integrated into a common reference frame [35,36] and it may be that the inconsistency of these signals in vection could introduce noise in that transformation. For instance, noise could be introduced in estimates of the body’s position in space and in spatial updating, due to vection itself. Somatosensory cues—such as pressure felt on the side of the body in the tilted posture—could also add further conflict and noise in resolving stimulus tilt relative to gravity. However, precision of judgements was higher in the self-motion compared to object-motion conditions. This seems to indicate that a different strategy is used in the estimation of self-motion direction or is possibly affected by the inconsistency of the visual and vestibular signals that occurs during vection.

### 4.2. Sensory Integration

A recent paper has modelled the effect of such inconsistent signals in judgements of haptic vertical by explicitly estimating the likelihood of multiple sensory signals arising from a common cause (the ‘casual inference’ model) [37]. If two sensory signals are commensurable and unbiased then the optimal combined estimator weights the cues according to their reliability; however, a robust estimator should not integrate widely discrepant cues as estimates will be biased and match neither sense [38]. The causal inference model [37] makes robust estimates of vertical by combining estimates from fused visual and vestibular signals with estimates from each sense alone, weighted in proportion to the probability the signals arose from a common cause or separate causes, respectively. Fused percepts should be less variable than single cue percepts even though bias will be greater when cues are discrepant. Consistent with this prediction, our data showed that smaller JNDs were associated with larger bias.

However, recall that in the present experiments that there were no informative cues to absolute orientation in the visual test stimuli themselves. Neither the line nor the optic flow indicated the direction of up. Even if there were a bias to see any line/flow as vertical this would have affected all the visual stimuli and not provided a reliable cue across trials with different tilts. Nevertheless, the differences in A-effect across our conditions may reflect the expected covariation of signals or the dependence on idiotropic vector. For instance, the onset of visual self-motion should be accompanied by an otolithic translation signal that should be fused with the visual signal. This may complicate parsing tilt from translation and reduce the reliability of the vestibular tilt signal. Further experimentation, independently controlling visual and vestibular static and dynamic orientation cues, would be helpful in determining the role of causal inference in these judgements.

### 4.3. Neural Processing Considerations

The differences in tilt biases under different conditions in our experiment may reflect the differences in neural processing required. The subjective visual vertical is disturbed in a wide range of neurological lesions and conditions [39] and cortical areas such as the insular cortex [40], inferior frontal gyrus and superior temporal gyrus [41] have been associated with perception of verticality. Lesions to thalamic and higher vestibular centres can disturb the subjective vertical without abnormal eye movements [39]. Cortical lesions involving the posterior insula are often associated with distortions of the subjective visual vertical [40] although these symptoms may require involvement of adjacent regions as well [42]. Based on clinical lesion studies Bronstein argued against a single internal representation of verticality [43] and recent reviews have suggested distinct functional roles for multisensory (vestibular) areas [44].

This variety of internal representations of verticality and diversity of processing may underlie the differences in bias we observed in different static and motion conditions. Vestibular cortical sensitivity and visual-vestibular integration are distributed across a network of areas [45,46] but the area around the posterior Sylvian fissure has been associated with vestibular sensitivity and multisensory integration. At least two distinct vestibular areas in this region have been identified in monkey: the polysensory parietoinsular vestibular cortex (PIVC) and the visual posterior Sylvian area (VPS) [45,47]. Homologous areas in humans are the PIVC and the posterior insular cortex (PIC) [44]. Visual and vestibular signal processing must dissociate self- from object-motion, perform coordinate transformations and disambiguate tilt relative to gravity from translation. PIVC neurons show selective response to linear acceleration, typically exhibiting a response to linear translation but not to static tilt relative to gravity [48,49]. Activity in PIVC is reportedly suppressed in response to visual motion [44,50] but activity in PIC is enhanced [44], while cortical motion sensitive areas exhibit a wide variety of vestibular influences [46]. These differences in response may be related to the need to distinguish visual object from self-motion and to disambiguate vestibular tilt from translation signals. Our 2D motion, 3D volumetric motion and self-motion conditions differ in the need or outcome of such processing and biases in verticality may reflect contribution of these different cortical areas and representations.

### 4.4. Conclusions

In summary, we have shown that when the whole body is tilted, both a static line and the direction of optic flow (whether it is planar or volumetric) are typically perceived as tilted in the direction of the body tilt, demonstrating the A-effect. Additionally, tilting the body also typically results in visually-induced illusory self-motion exhibiting a similar but larger A-effect. We found systematic errors across all of the stimulus conditions when observers were tilted and this is in line with the notion that perceived line and motion axis tilt may be due to misestimations in self-tilt. However, the different magnitude A-effects for the line and motion conditions—and for object and self-motion—are not consistent with adding a head centric visual tilt estimate to a fixed but biased self-tilt estimate. This stimulus dependence may be due to differences in errors in combining coordinate frame transformations (eye to head to allocentric) for visual signals with different reliability or due to differences in processing inconsistencies in the idiotropic and vestibular signals for different visual signals, particularly so in the case of vection which occurs despite visual-vestibular conflict. Further research into the integration of other visual cues such as lighting, could help provide insight into how noise might be reduced in the determination of tilt of external motion and perceived self-motion.
